# Bilateral Pleural Effusions due to Pulmonary Amyloidosis as the Presenting Manifestation of Multiple Myeloma

**DOI:** 10.4084/MJHID.2012.010

**Published:** 2012-01-25

**Authors:** Abhishek Agarwal, Sandeep Singla, Meghana Bansal, Bijay Nair

**Affiliations:** 1 Department of Internal Medicine, University of Arkansas for Medical Sciences, Little Rock, USA; 2 Myeloma Institute of Research and Therapy, University of Arkansas for Medical Sciences, Little Rock, USA

## Abstract

Multiple Myeloma is a hematologic malignancy of plasma cell origin. Pleural effusion may develop in the setting of myeloma due to various reasons but is extremely uncommon as a presenting symptom. A 69-year-old Caucasian man presented with pleural effusions of undetermined etiology after extensive work up, and multiple failed pleurodesis. Lung biopsy revealed pulmonary amyloidosis and led to the diagnosis of multiple myeloma. Patient was started on chemotherapy but died within 6 weeks of his diagnosis due to multiorgan failure. Pulmonary amyloidosis should be suspected as a cause of intractable pleural effusions, even in patient who do not have evidence of lung involvement on imaging studies or typical features of multiple myeloma. Pleural effusions due to amyloidosis are often refractory to treatment, and a high index of suspicion is required for early diagnosis and treatment.

## Introduction

Though a pleural effusion occurs in approximately 6% of patients with Multiple Myeloma (MM) during the course of the disease, it is not usually a presenting sign.^1^,^2^ We present a case of a 69-year-old Caucasian man who presented with intractable bilateral pleural effusions for 2 years without a clear etiology despite extensive work up at other centers. Our patient did not have the typical features of MM such as bone lesions, hypercalcemia or renal failure at the time of diagnosis. During the course of his work up, he was noted to have pulmonary amyloidosis on a lung biopsy, and further investigations led to an unsuspected diagnosis of MM. MM should be considered in the differential of intractable pleural effusions and testing for paraproteins should be considered. Also, this case highlights the point that a lung biopsy should be considered early in the work up of recurrent pleural effusions when a diagnosis is not forthcoming on usual investigations, even if the lung parenchymal appears to be uninvolved on imaging studies.

## Case report

A 69-year-old man presented with recurrent bilateral pleural effusions of unclear etiology. He had undergone thoracocentesis more than 25 times over the last 2 years and had bilateral pleurodesis multiple times without success. Pleural fluid studies and a pleural biopsy were negative for malignancy or infection. He had no history of chest trauma, thoracic surgery, tuberculosis, lymphoma, cancer or significant exposure to asbestos. His renal, hepatic and cardiac function had been normal. He had smoked one-half pack cigarettes a day for 40 years and quit a year ago. He reported a chronic cough, exertional dyspnea, severe anorexia and weight loss of 30 kilograms in last 3 months. Due to his intractable pleural effusions of undetermined etiology, he was admitted for a lung biopsy and ligation of thoracic ducts.

**Figure 1:**
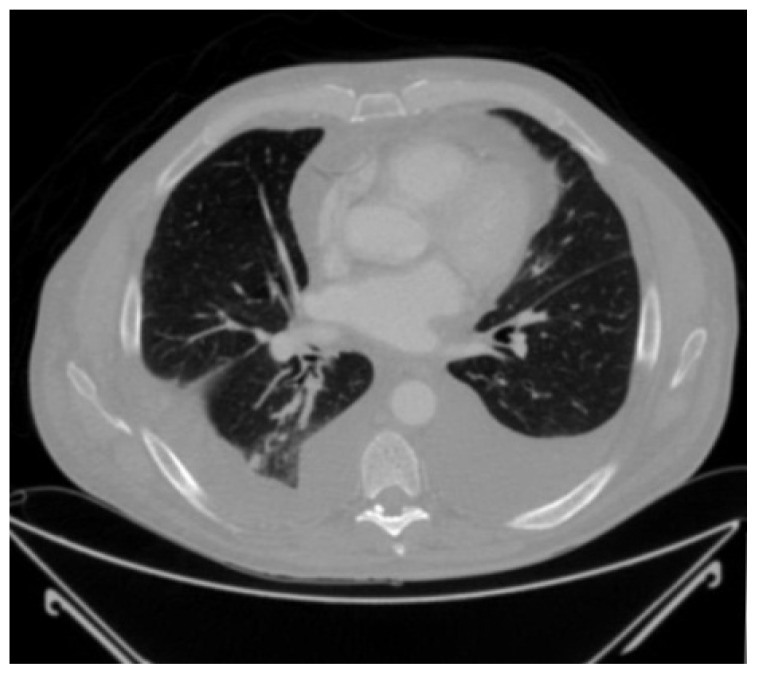
CT chest showing bilateral pleural effusions and clear lung parenchyma.

The physical examination revealed a man with mild shortness of breath. He had generalized edema, decreased breath sounds over the lung bases and a normal cardiac examination. He was afebrile with normal respirations, a pulse of 86 beats per minute and blood pressure of 145/79 mm of Hg. White blood count was 8700 cells/mm^3^, hemoglobin was 10 g/dL, serum creatinine was 1.1 mg/dL and serum calcium was 8.8 mg/dL. Serum protein was 3.7 g/dL with albumin of 2.7 g/dL. Liver function tests were normal. Urinalysis showed 30 mg/dL protein in a spot sample. C-reactive protein was elevated at 50 mg/L. Anemia work up was consistent with anemia of chronic disease with normal serum B12 and folate levels. A chest x-ray showed bilateral pleural effusions. Echocardiogram showed normal left ventricular ejection fraction of 60% and there were no features of cardiac amyloidosis. Computed tomography of the chest and abdomen showed multiple calcified mediastinal lymphadenopathy and pleural effusions with atelectasis of the lung bases, but no obvious parenchymal lung lesions (Figure 1).

**Figure 2:**
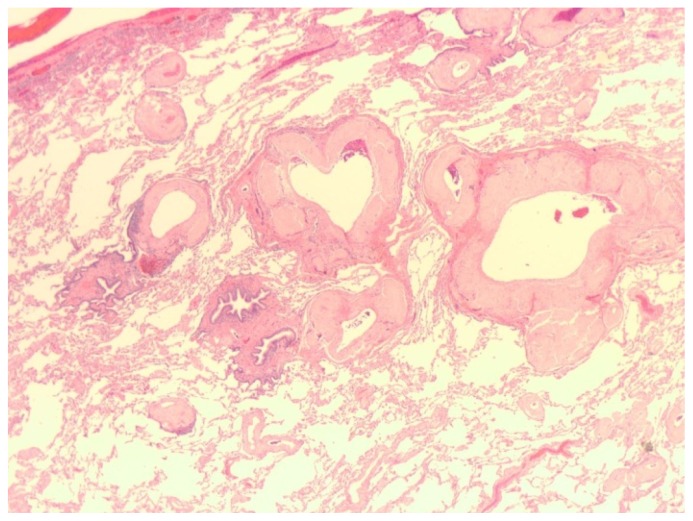
Lung biopsy shows hyaline amyloid deposits, predominantly in a perivascular pattern.

Pleural fluid was exudative with a white count of 212 cells/mm^3^ with 88% lymphocytes. Fluid triglycerides were elevated at 277 mg/dl, lactate dehydrogenase was 133 IU/L and fluid protein was 2800 mg/dL. The fluid was negative for infection, plasma cells or other tumor cells. Histopathology of biopsy of the right lung revealed pulmonary amyloidosis, mostly in a perivascular pattern, seen as hyaline deposits on Hematoxylin & Eosin stain (Figure 2). Apple-green birefringence was noted on Congo-red stain under polarized light. Mass spectroscopic subtyping revealed AL-kappa amyloid deposits.

**Figure 3:**
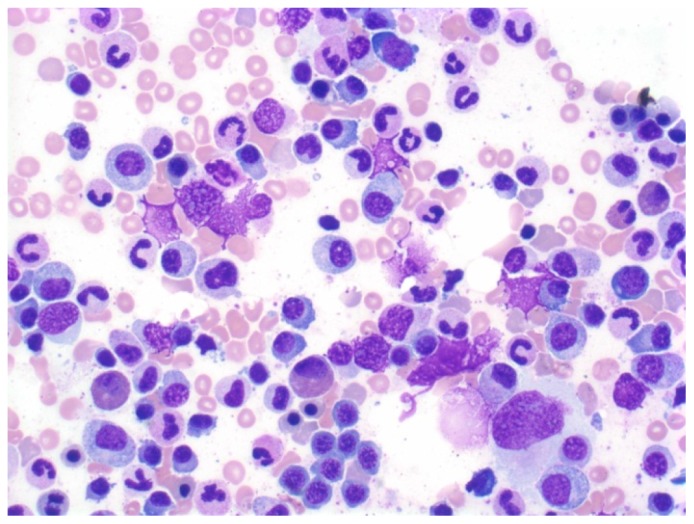
Bone marrow biopsy shows abundant atypical plasma cells with immature nuclei

Serum protein electrophoresis showed total protein of 3.3 g/dL with albumin 1.6 g/dL but absence of M-protein. Serum and urine immunofixation revealed presence of kappa free light chains. Serum free light chain analysis showed kappa free light chains of 47.2 mg/dL (ref 0.33–1.95 mg/dL) and a kappa to lambda light chain ratio of 41.4 (ref 0.26–1.65). Urine protein electrophoresis showed 24-hour urine protein of 1201 mg with M-protein of 40%. A bone marrow biopsy revealed 25% plasma cells comprised predominantly of kappa-expressing cells (Figure 3). A skeleton survey did not show any lytic or blastic lesions.

Our patient met the diagnostic criteria for MM based on bone marrow biopsy showing more than 10% plasma cells, monoclonal protein in the serum and urine, and presence of a myeloma-related organ dysfunction in the form of anemia. The etiology of his intractable effusion was felt to be pulmonary amyloidosis secondary to multiple myeloma. He did not have features of amyloidosis elsewhere such as cardiac failure, hepatomegaly, nephrotic syndrome and peripheral neuropathy. He was started on melphalan and bortezomib. His pleural effusions persisted despite thoracic duct ligation. His hospital course was complicated with acute renal failure, severe anasarca, deep vein thrombosis, congestive heart failure, myocardial infarction and sepsis. The diagnosis of MM as the cause of his intractable pleural effusion was terminal as the patient expired within a few weeks of diagnosis.

## Discussion

MM accounts for 1% of all cancers and 10% of all hematologic malignancies.^3^ It mainly affects the bone marrow, but may involve other organ-systems. Thoracic manifestations include osseous lesions, plasmocytoma, pulmonary infiltrates, mediastinal lymphadenopathy and pleural effusion.^1^,^4^ In patients with MM, clinically evident primary amyloidosis develops in the course of disease in 10% to 15% of patients.^5^ Pulmonary amyloidosis may present as pulmonary nodules, diffuse interstitial amyloidosis, or pleural effusion associated with pleural amyloid deposition.^6^

Pleural effusion in MM may occur due to several mechanisms: namely, an involvement of the pleural space from adjacent skeletal or parenchymal tumors, mediastinal lymph node infiltration with lymphatic obstruction, and pleural or pulmonary amyloidosis.^1^,^4^,^7^ MM can be complicated by nephrotic syndrome, chronic renal failure, restrictive amyloid cardiomyopathy, and pulmonary embolism, which can all result in pleural effusion. Direct myeloma involvement of the pleural space is extremely rare occurring in < 1% of cases.^8^ Infectious etiology of effusion is common due to associated hypogammaglobulinemia. Large persistent pleural effusions refractory to diuretics and thoracentesis are more likely to be due to pleural amyloid infiltration.^9^ In our case, the pleural fluid cytology did not reveal any myelomatous cell, and the recurrent effusions were secondary to pulmonary amyloidosis.

Light chain myeloma can be missed on serum electrophoresis, as in our case, and urine electrophoresis should always be done in the work up of MM. Pulmonary amyloidosis can be diagnosed by bronchoscopic lung biopsy with reasonable safety; although the bronchoscopist should be prepared to manage hemorrhage after biopsy.^10^ Pleural involvement can be diagnosed by presence of plasma cells in pleural fluid or a pleural biopsy.

Pleural effusions due to amyloidosis or myelomatous involvement of the pleural space are often refractory to treatment and carry a poor prognosis. Escalaton of testing should be considered early in the work up of recurrent pleural effusion when diagnosis remains unclear on less invasive testing, as it can prevent delays in diagnosis and treatment.
